# Do electronic reminders alter recorded diagnoses in primary care office-hours practices of health centers: A register-based study in a Finnish city

**DOI:** 10.1177/20503121211036117

**Published:** 2021-07-29

**Authors:** Mika Lehto, Kaisu Pitkälä, Ossi Rahkonen, Merja K Laine, Marko Raina, Timo Kauppila

**Affiliations:** 1 Department of General Practice and Primary Health Care, University of Helsinki, Helsinki, Finland; 2 Unit of Primary Health Care, Helsinki University Hospital, Helsinki, Finland; 3 Vantaa Social and Health Bureau, Vantaa, Finland; 4 Department of Public Health, University of Helsinki, Helsinki, Finland; 5 Folkhälsan Research Centre, Helsinki, Finland

**Keywords:** Diagnose, electronic medical record, electronic reminder, primary care, recording

## Abstract

**Objectives:**

One purpose of electronic reminders is improvement of the quality of documentation in office-hours primary care. The aim of this study was to evaluate how implementation of electronic reminders alters the rate and/or content of diagnostic data recorded by primary care physicians in office-hours practices in primary care health centers.

**Methods:**

The present work is a register-based longitudinal follow-up study with a before-and-after design. An electronic reminder was installed in the electronic health record system of the primary health care of a Finnish city to remind physicians to include the diagnosis code of the visit in the health record. The report generator of the electronic health record system provided monthly figures for the number of various recorded diagnoses by using the International Classification of Diseases, 10th edition, and the total number of visits to primary care physicians, thus allowing the calculation of the recording rate of diagnoses on a monthly basis. The distribution of diagnoses before and after implementing ERs was also compared.

**Results:**

After the introduction of the electronic reminder, the rate of diagnosis recording by primary care physicians increased clearly from 39.7% to 87.2% (p < 0.001). The intervention enhanced the recording rate of symptomatic diagnoses (group R) and some chronic diseases such as hypertension, type 2 diabetes and other soft tissue disorders. Recording rate of diagnoses related to diseases of the respiratory system (group J), injuries, poisoning and certain other consequences of external causes (group S), and diseases of single body region of the musculoskeletal system and connective tissue (group M) decreased after the implementation of electronic reminders.

**Conclusion:**

Electronic reminders may alter the contents and extent of recorded diagnosis data in office-hours practices of the primary care health centers. They were found to have an influence on the recording rates of diagnoses related to chronic diseases. Electronic reminders may be a useful tool in primary health care when attempting to change the behavior of primary care physicians.

## Introduction

Electronic reminders have been used in primary health care because of their effectiveness in modifying the clinical practice of physicians.^1^ Electronic reminders have been applied in order to alter the clinical decision making of primary care physicians (PCPs), for example, general practitioners and other physicians working in primary health care. Electronic reminders have been used to enhance the use of certain medications^2^ and implementing screening^3^ in accordance with clinical guidelines. They have been reported to have a positive impact when, for example, trying to reduce the excessive use of antibiotics to treat throat infections^4^ or screening for diabetic retinopathy performed^5^ according to the appropriate guidelines. In order to enhance the preventive work of PCPs, electronic reminders have been implemented to promote the administration of vaccinations.^6^ Not all interventions with electronic reminders have been successful, or have not improved the patient outcome when treating, for example, angina, asthma, chronic obstructive lung disease or hypertension.^7–9^ Therefore, the best design for electronic reminders is not known. The most appropriate targets for their implementation are not clear, either.^7–9^

Since the quality of data in the electronic health record (EHR) is only as reliable, and therefore as useful, as the quality of the data that are entered into it by the personnel of health care facilities,^10^ one very important function of electronic reminders is improving the quality of documentation in PCPs’ clinical practice. Yet, to the best of our knowledge, no studies have been carried out to identify the impact of electronic reminders on the quality of documentation in EHRs. Electronic reminders lead to an increase in the rate at which diagnoses are recorded in primary health care visits to PCPs.^11,12^ Recording diagnoses in primary care is not self-evident because in 2018, less than two-thirds of the visits to the Finnish PCPs contained a recorded diagnosis.^13^ Thus, it seemed reasonable to study the relationship of electronic reminders to the contents and quantity of documentation in office-hours primary care health centers (PCHCs). Entering an appropriate diagnosis code into the EHR is crucial for various computerized systems to support clinical decision making.^2,4,5^ Thus, the primary aim of this study was to evaluate whether implementation of electronic reminders alters the rate and/or content of diagnostic data recorded by office-hours PCPs in PCHCs.

## Materials and methods

This is a register-based longitudinal follow-up study with a before-and-after design in the primary care of the fourth-most-populated city of Finland. The study was performed in the primary health care of the city of Vantaa, having about 200,000 inhabitants in the year 2008. There were 123 PCPs in 2002 when the study began, and at the lowest level, in 2007, there were 106. The number of PCPs increased to 130 at the end of the study in 2014. The Finnish primary health care and the EHR systems used in Vantaa, as elsewhere in Finland, are mainly maintained by municipalities funded with tax income.

The study was carried out by examining data from the EHR without identifying the patients or PCPs. The register holder (the health authorities of Vantaa) and the scientific ethical board of the city of Vantaa (TUTKE) granted permission (VD/8059/13.00.00/2016) to carry out the study and waived the requirement for written informed consent from the subjects.

The data of the PCHCs of the Vantaa City were obtained from the Graphic Finstar EHR system (GFS, Logica LTD, Helsinki, Finland). GFS provides a specific field in the EHR where an appropriate diagnosis code (based on the 10th edition of the International Classification of Diseases (ICD-10)) can be entered during the patient’s visit to the office-hours PCP in the PCHC. The EHR system assists the PCP in assigning an appropriate diagnosis code or allows the physician to enter the desired diagnosis code to the system directly as described in detail earlier.^11,12^ As a result, it takes between 2 and a few dozen seconds longer for the PCP to record the visit or consultation.

The report generator of the GFS system provided monthly figures for the number of different recorded diagnoses and the total number of office-hours PCP visits, thus allowing the calculation of the recording rate of each diagnosis as a percentage of total visits on a monthly basis without identifying individual PCPs or patients. For analysis, the ICD-10 diagnoses were examined using the first letter or three first characters. Distributions of the diagnoses recorded in the office-hours PCP practice were the primary measure for analysis in the present study. The 20 most commonly recorded diagnoses were analyzed in more detail. The proportion of the visits having a recorded diagnosis in the office-hours PCP practice was a secondary measure.

On 1 February 2008, an electronic reminder was installed into the GFS system. This intervention cost less than 10,000 €. After installation, the reminder remained active until the end of our study on 31 December 2014. The GFS system prompted PCPs to enter a diagnosis code every time they wanted to complete the recording of the visit.^11,12^ The follow-up period started from February 2002 and ended in December 2014.

### Statistical methods

The obtained data were analyzed by comparing the rates and proportions of the 20 most frequently recorded diagnoses during 6-year periods before (2002–2007) and after (2009–2014) the installation of the electronic reminder into the EHR system (1 February 2008) of the primary health care system in the city of Vantaa, Finland. These comparisons were performed with t-test or Mann–Whitney U test, when appropriate. The proportion of visits having recorded diagnoses during the follow-up before (2002–2007) and after (2009–2014) the intervention were compared using repeated-measures analysis of variance (RM-ANOVA), followed by Bonferroni correction.

## Results

### General diagnostics

**Figure 1.**
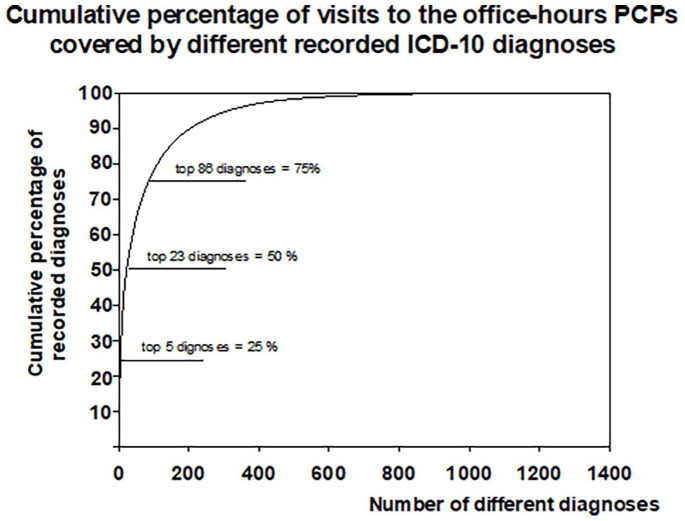
Cumulative percentage of visits to the office-hours primary care physicians (PCPs) as a function of different recorded diagnoses by the 10th edition of the International Classification of Diseases in the city of Vantaa, Finland.

During the study, there were 2,473,715 visits to the office-hours primary care PCPs in the PCHCs and the total number of visits having recorded diagnoses was 1,527,867 (61.7%). Altogether, 1586 different ICD-10 diagnoses were assigned. The 20 most common diagnoses were recorded for 43% of the visits in which a diagnosis was recorded (Figure 1) and 26.5% of all the recorded visits. The most common recorded diagnoses were acute upper respiratory infections, back pain, suppurative and unspecified otitis media, acute sinusitis, acute bronchitis, and essential (primary) hypertension (Table 1).

**Table 1.** The distribution of the 20 most common International Classification of Diseases, 10th edition (ICD-10), diagnoses made by the office-hours primary care physicians during the follow-up (2002–2014) in the city of Vantaa, Finland.

ICD-Code	Diagnosis, Office-hours primary care physicians	N	% of visits with diagnosis	% of all visits
J06	Acute upper respiratory infections of multiple and unspecified sites	142,039	9.30%	5.74%
M54	Back pain	81,174	5.31%	3.28%
H66	Suppurative and unspecified otitis media	60,288	3.95%	2.44%
J01	Acute sinusitis	50,089	3.28%	2.02%
J20	Acute bronchitis	48,219	3.16%	1.95%
I10	Essential (primary) hypertension	47,373	3.10%	1.92%
R10	Abdominal and pelvic pain	34,660	2.27%	1.40%
E11	Type 2 diabetes mellitus	32,740	2.14%	1.32%
H10	Conjunctivitis	32,406	2.12%	1.31%
F32	Depressive episode	27,040	1.77%	1.09%
M79	Other soft tissue disorders, not elsewhere classified	25,877	1.69%	1.05%
M17	Gonarthrosis [arthrosis of knee]	23,721	1.55%	0.96%
M75	Shoulder lesions	22,843	1.50%	0.92%
J45	Asthma	20,524	1.34%	0.83%
R05	Cough	16,979	1.11%	0.69%
M53	Other back pains, not elsewhere classified	15,269	1.00%	0.62%
A09	Other gastroenteritis and colitis of infectious and unspecified origin	13,994	0.92%	0.57%
F41	Other anxiety disorders	13,847	0.91%	0.56%
H60	Otitis externa	13,090	0.86%	0.53%
J03	Acute tonsillitis	12,916	0.85%	0.52%

### Effects of electrical reminders on diagnostics

The absolute numbers of recorded diagnoses increased after the implementation of the electronic reminders and remained elevated (Tables 2 and 3). The percentage of recorded diagnoses in the office-hours practice increased after the introduction of electronic reminders from 39.7% (SD 1.6, 2002–2007 before the intervention) to 87.2% (SD 4.9, 2009–2014 after the intervention; p < 0.001, RM-ANOVA). The proportion of various mild infectious diseases, such as acute upper respiratory infections, decreased (11.1% ⩾ 8.14%, p < 0.001) after implementing electronic reminders. Conversely, the recording rate of certain chronic diseases, such as hypertension (2.68% ⩾ 3.33%), type 2 diabetes (1.35% ⩾ 2.54%), anxiety (0.74% ⩾ 0.99%) and arthrosis of knee (0.88% ⩾ 1.89%) was enhanced (all p < 0.001, Table 2). Recording of symptomatic diagnoses, such as abdominal and pelvic pain (1.88% ⩾ 2.47%) or cough (0.99% ⩾ 1.19%), was enhanced (p < 0.001).

**Table 2.** Comparison of percentages and absolute numbers of different International Classification of Diseases, 10th edition (ICD-10), diagnosis groups 6 years before (2002–2007) and 6 years after (2009–2014) the implementation of electronic reminders in the office-hours primary care during the follow-up (2002–2014) in the city of Vantaa, Finland.

ICD-Code	Diagnosis set by the office-hours primary care physicians	Before electronic reminders, (%/year) % of recorded diagnoses	After electronic reminders, (%/year) % of recorded diagnoses	Before electronic reminders, (%/year), % of all visits	After electronic reminders, (%/year) % of all visits	Before electronic reminders (N/year)	After electronic reminders, (N/year)
J06	Acute upper respiratory infections of multiple and unspecified sites	11.1 (10.5–13.0)	8.14 (7.66–8.44)**	4.58 ± 0.38	7.04 ± 0.35***	8975 ± 790	12,845 ± 832***
M54	Back pain	6.26 ± 0.25	4.81 ± 0.16***	2.50 ± 0.17	4.19 ± 0.21***	4884 ± 331	7636 ± 434***
H66	Suppurative and unspecified otitis media	4.82 ± 0.46	3.47 ± 0.32***	1.92 ± 0.17	3.02 ± 0.28***	3760 ± 353	5514 ± 575***
J01	Acute sinusitis	4.63 ± 0.56	2.59 ± 0.44***	1.84 ± 0.17	2.24 ± 0.28*	3599 ± 355	4084 ± 523
J20	Acute bronchitis	4.30 ± 0.48	2.55 ± 0.7***	1.65 (1.58–1.90)	2.32 (1.61–2.65)	3312 (3074–3668)	4212 (2941–4791)
I10	Essential (primary) hypertension	2.68 ± 0.24	3.33 ± 0.09***	1.07 ± 0.13	2.90 ± 0.13***	2093 ± 248	5278 ± 271***
R10	Abdominal and pelvic pain	1.88 ± 0.13	2.47 ± 0.09***	0.75 ± 0.08	2.16 ± 0.2***	1467 ± 143	3934 ± 388***
E11	Type 2 diabetes mellitus	1.35 ± 0.39	2.52 ± 0.14***	0.54 ± 0.17	2.20 ± 0.20***	1061 ± 341	4006 ± 385***
H10	Conjunctivitis	2.49 ± 0.25	1.91 ± 0.28**	0.99 ± 0.13	1.66 ± 0.25***	1606 ± 163	2558 ± 154***
F32	Depressive episode	2.06 ± 0.18	1.62 ± 0.18**	0.82 ± 0.09	1.40 ± 0.08***	1941 ± 246	3029 ± 477***
M79	Other soft tissue disorders, not elsewhere classified	1.10 (0.96–1.21)	1.87 (1.61–2.38)**	0.42 (0.39–0.49)	1.65 (1.33–2.16)*	839 (731–980)	3031 (2409–3912)**
M17	Gonarthrosis (arthrosis of knee)	0.88 ± 0.27	1.89 ± 0.3***	0.35 *±* 0.12	1.66 *±* 0.33***	692 ± 234	3019 ± 615***
M75	Shoulder lesions	1.55 ± 0.06	1.47 ± 0.1	1.55 ± 0.06	1.47 ± 0.1	1213 ± 83	2337 ± 253***
J45	Asthma	1.29 ± 0.06	1.38 ± 0.08	0.52 ± 0.03	1.20 ± 0.05***	1010 ± 61	2185 ± 116***
R05	Cough	0.99 ± 0.09	1.19 ± 0.07*	0.39 ± 0.03	1.04 ± 0.11***	771 ± 64	1898 ± 205***
M53	Other back pains, not elsewhere classified	1.28 (1.24–1.35)	0.85 (0.83–0.86)**	0.52 ± 0.04	0.74 ± 0.05***	1013 ± 76	1340 ± 90***
A09	Other gastroenteritis and colitis of infectious and unspecified origin	1.19 (1.15–1.39)	0.70 (0.68–0.76)**	0.49 (0.46–0.54)	0.62 (0.6–0.66)	954 (881–1076)	1118 (1105–1182)
F41	Other anxiety disorders	0.74 ± 0.09	0.99 ± 0.04***	0.3 ± 0.04	0.86 ± 0.08***	579 ± 88	1569 ± 162***
H60	Otitis externa	0.96 (0.85–1.11)	0.79 (0.76–0.86)*	0.39 ± 0.05	0.70 ± 0.06***	757 ± 98	1276 ± 129***
J03	Acute tonsillitis	1.03 (0.9–1.40)	0.66 (0.54–0.85)**	0.45 ± 0.08	0.59 ± 0.1	887 ± 160	1073 ± 183

The data are expressed as mean ± standard deviation or median (25%–75% quartile range).

* stands for p < 0.05, **p < 0.01 and ***p < 0.001 before versus after, t-test or Mann–Whiney U test when appropriate.

**Table 3.** The distribution of the main groups of 10th edition of the International Classification of Diseases (ICD-10) diagnoses before (2002–2007) and after (2009–2014) application of electronic reminders in the office-hours primary health care in the city of Vantaa, Finland.

ICD-10 letter	Contents of diagnosis group	% of all diagnoses		% of all visits	
		Before electronic reminder	After electronic reminder	Before electronic reminder	After electronic reminder
A	Intestinal infectious diseases, bacterial infections and viral infections of central nervous system	1.85 (1.77–2.06)	1.28 (1.26–1.31)**	0.77 ± 0.1	1.28 ± 0.04***
B	Other infections	1.29 ± 0.08	1.13 ± 0.13*	0.51 ± 0.02	1.13 ± 0.13***
C	Malignant neoplasms	0.26 ± 0.4	0.37 ± 0.06**	0.10 ± 0.02	0.37 ± 0.06***
D	Other neoplasms and carcinoma in situ	0.44 ± 0.6	0.89 ± 0.23***	0.18 ± 0.03	0.89 ± 0.23***
E	Endocrine nutritional and metabolic diseases	2.66 ± 0.63	3.66 ± 0.26**	1.07 ± 0.29	3.66 ± 0.26***
F	Mental and behavioral disorders	5.15 (4.42–5.28)	4.76 (4.20–4.87)	1.97 ± 0.25	4.60 ± 0.37***
G	Diseases of the nervous systems	1.99 ± 0.09	1.61 ± 0.28**	0.8 ± 0.04	1.61 ± 0.28***
H	Diseases of the eye and the adnexa, and the ear and mastoid process	10.35 ± 0.68	7.76 ± 0.75***	4.13 ± 0.35	7.76 ± 0.75***
I	Diseases of the circulatory system	5.72 (4.51–5.83)	6.15 (5.68–6.33)	2.14 ± 0.33	6.04 ± 0.36***
J	Diseases of the respiratory system	27.44 ± 2.92	16.39 ± 1.11***	10.9 ± 0.8	16.39 ± 1.11***
K	Diseases of the digestive system	2.77 ± 0.23	2.41C0.2***	1.11 ± 0.13	2.41 ± 0.2***
L	Diseases of the skin and subcutaneous tissue	3.90 ± 0.22	4.55 ± 0.61*	1.56 ± 0.12	4.55 ± 0.61***
M	Diseases of the musculoskeletal system and connective tissue	17.41 ± 0.89	15.11 ± 1.41**	6.94 ± 0.59	15.12 ± 1.41***
N	Diseases of genitourinary system	2.12 ± 0.14	2.6 ± 0.25**	0.84 ± 0.07	2.6 ± 0.25***
O	Pregnancy, childbirth and puerperium	0.54 ± 0.07	0.33 ± 0.04***	0.22 ± 0.04	0.33 ± 0.04***
P	Certain conditions originating in the perinatal period	0.012 ± 0.005	0.006 ± 0.002*	0.005 ± 0.002	0.006 ± 0.002
Q	Congenital malformations, deformations and chromosomal abnormalities	0.14 ± 0.02	0.13 ± 0.02	0.06 ± 0.01	0.13 ± 0.02***
R	Symptoms, signs and abnormal clinical and laboratory findings, not elsewhere classified	7.52 ± 0.38	9.48 ± 0.76***	3.00 ± 0.25	9.48 ± 0.76***
S	Injury, poisoning and certain other consequences of external causes, single body region	6.1 ± 0.33	4.18 ± 0.56***	2.43 ± 0.15	4.18 ± 0.56***
T	Injuries to multiple or unspecified body regions as well as poisoning and certain other consequences of external causes.	0.69 ± 0.04	0.68 ± 0.03	0.28 ± 0.02	0.68 ± 0.03***
V	Transport accidents	0.006 ± 0.003	0.003 ± 0.002	0.002 ± 0.001	0.003 ± 0.002
W	Other external causes of accidental injury	0.12 ± 0.03	0.18 ± 0.02**	0.05 ± 0.01	0.18 ± 0.02***
X	Exposure to burning substances and related threads, venomous animals and plants, noxious substances and forces of nature. Intentional self-harm and assault	0.008 ± 0.002	0.011 ± 0.003	0.003 ± 0.001	0.011 ± 0.003***
Y	Events of undetermined intent, legal interventions and operations of war, complications of medical care, sequelae of external causes of morbidity and mortality	0.011(0.008–0.018)	0.021(0.019–0.031)**	0.004 ± 0.003	0.021 ± 0.019**
Z	Factors influencing health status and contact with health services	2.00 ± 0.36	3.65 ± 0.80***	0.8 ± 0.13	3.65 ± 0.80***

The data are expressed as mean ± standard deviation or median (25%–75% quartile range).

* stands for p < 0.05, **p < 0.01 and ***p < 0.001 before versus after, t-test or Mann–Whiney U-test when appropriate.

Those ICD-10 diagnosis groups which were the most frequently recorded diagnoses before application of electronic reminders decreased in their relative proportions of diagnoses (p < 0.001, Table 3). These included diseases of the respiratory system (group J, 27.44% ⩾ 16.39%), injuries, poisoning and certain other consequences of external causes, single body region (group S, 6.1% ⩾ 4.18%), and diseases of the musculoskeletal system and connective tissue (group M, 17.41% ⩾ 15.11%). The only exception was group R, symptoms, signs and abnormal clinical and laboratory findings (not elsewhere classified) whose proportion increased (7.52% ⩾ 9.48%, p < 0.001).

## Discussion

Most of the recorded diagnoses in the office-hours PCP practices of the PCHCs were infections in the upper part of the respiratory system both before and after the introduction of electronic reminders. Electronic reminders had mostly a facilitating impact in the recording of diagnoses of chronic diseases. The recording rate of symptomatic diagnoses, ICD-10 code group R diagnoses, was also enhanced while the proportion of some others, such as group J diagnoses, diseases of the respiratory system, group S, injuries, poisoning and certain other consequences of external causes, and group M, diseases of single body region of the musculoskeletal system and connective tissue, decreased after the implementation of electronic reminders. Thus, the effect of electronic reminders on the recording of diagnoses in the office-hours PCP practices seemed to be variable: recording rates of some diagnoses increased and some others did not.

From our former study, we already knew that the application of electronic reminders in the EHR increased the recording rate of diagnoses and that the distribution of diagnoses from the last year of that follow-up (2014) was similar to the distribution of recorded diagnoses reported from other units of Finnish primary care.^12^ In the present study, prompting the recording of missing diagnosis data with electronic reminders altered not only the volume but also the distribution of recorded diagnoses in the office-hours practices of the PCHCs. PCPs increased the recording of ICD-10 system-based diagnoses of certain chronic diseases. According to our study observations, the relative proportion of the recorded diagnoses of some of the chronic diseases, such as hypertension, type 2 diabetes, miscellaneous connective tissue disorders and osteoarthrosis of knee, increased after the introduction of electronic reminders while some others, such as depression, asthma and back pains, did not. Proportional recordings of some chronic diagnoses, such as depression and back pain, were not increased in the same way as some others. However, the burden of disease induced by these diagnoses is certainly not decreasing.^14^ A putative explanation is that the recording of these diseases was already fairly frequent before application of electronic reminders and so few people with depression or back pain was uncoded. After intervention, there was an increase in other, less well-recorded diagnoses. Thus, the decrease in proportions of depression or back pain diagnoses is related to the denominator increasing. Nevertheless, further, possibly qualitative, studies with local PCPs should be carried out to understand why the recording was performed in the way it was. Documentation of chronic diseases might theoretically serve as one of the first targets in improving the quality of care.^15,16^ Due to its importance, improving the level of this documentation using interventions is worth aiming for.^17^ However, not all interventions, for example, financial incentives, are necessarily very successful in enhancing the documentation of chronic diagnoses, such as type 2 diabetes.^17^

In addition, there was an increase in diagnosis codes referring to so-called symptomatic diagnoses: diagnoses describing only the symptoms, signs or abnormal clinical findings while not suggesting any specific illnesses underlying these symptoms.^18^ Thus, the PCPs may not have reached a conclusion in terms of a specific diagnosis in all situations. As has been reported before,^19,20^ this is common with unscreened patients and therefore diagnosis recordings may have been neglected to some extent before the present intervention. Upon prompting the recording of the missing diagnosis documentation with electronic reminders, physicians were more inclined to adapt to recording symptoms using the ICD-10 system.

As stated in the introduction, there are occasions when electronic reminders in primary care have been proved to be useful^2–6^ and situations when they were not.^7–9^ There seems to be some logic in whether or not PCPs follow instructions given via electronic reminders. For example, when an electronic reminder suggests potentially useful vaccinations they are complied with very well.^21^ Analogously, simple screening procedures, such as screening for colorectal cancer, are performed after an electronic reminder but a little less eagerly.^22^ However, if there is a bigger decision to be made to guide long-term treatment of a patient, for example, deciding whether or not to start continuous preventive anticoagulation, the reminders are less eagerly complied with by PCPs.^23^ From a technical point of view, our intervention with reminders was directed toward a minor procedure, recording a diagnosis. Therefore, reminders proved to be successful in enhancing the number of recorded diagnoses. However, the complexity involved in determining an accurate diagnosis resulted in the high percentage of symptomatic diagnoses recorded. This uncertainty is also one factor which might have caused the observed changes in the distribution of recorded diagnoses.

Yet there may also have been secular trends affecting the observed change in the distribution of diagnosis recordings. The decrease in relative proportions of diagnosis recordings representing mild infections, depression and back pain may have been related to changes in the selection of patients scheduled for office-hours visits in the PCHCs. Nor do we know whether electronic reminders were solely responsible for any change in practice, or how far the increased recording was due to education and feedback given to the physicians in the primary care of the city of Vantaa at the time the electronic reminder was applied.^11,12^ It is well known that auditing and feedback influence the activities of physicians,^24^ including in primary care.^25,26^ However, there were no changes in the primary care office-hours system during the study and there was considerable variation in the amount and frequency of feedback given^11,12^ whereas the reminder was introduced systematically and simultaneously to all users in 2008. The change in recording diagnoses was abrupt and happened right after the electronic reminder was introduced. Thus, use of reminders in the present context seemed to have much larger impact than when they are used in guiding testing and prescribing.^1^ Therefore, it is fair to conclude that the reminder played a large role considering the fact that the diagnosis recording rate remained elevated throughout the remainder of the follow-up period.

There was also considerable variation in the percentages of diagnosis specific visits depending on whether the percentage was calculated using the number of all visits having a recorded diagnosis or all visits to the PCPs as a numerator. For example, in cases of acute upper respiratory infections, back pain, otitis media, acute sinusitis, bronchitis, depression and conjunctivitis, the percentage proportions calculated for these diseases decreased after implementation of electronic reminders when using visits having recorded diagnosis as a numerator but increased if the comparison was made with respect to all visits, regardless of the presence of diagnosis information. One possible explanation for these discrepancies may be that the overall number of visits to the office-hours PCPs decreased during the follow-up period in the city of Vantaa.^27^ Decreasing visits to PCPs has been a consistent and general trend in Finnish primary health care, while the precise reasons for this remain unknown.^28,29^ This decrease affected the percentage counted from all visits considerably, but not that counted from visits with recorded diagnoses. Therefore, studying diagnosis recordings as a measure of function should always be interpreted cautiously and by following several parameters of prevalence instead of observing only a single variable.

The strength of this study is that the present data reflect the “real life” activity of office-hours PCPs in PCHCs. The participants were unaware of being studied. The change in the rate of recording diagnoses was so rapid and large that it could hardly be due to secular trends.

There are several limitations in this study. The present results are only applicable to primary health care. As this was a cohort study in a community of about 200,000 people, it was not possible to calculate statistical power for the sample size. The sizes of the groups were random and no additional data were available. As a compromise, the analysis was done at a three-digit aggregated level of the ICD-10 system. Most certainly, this method of grouping affected the present results but we had to compromise to keep the sizes of the different diagnosis groups adequate for statistical comparisons. Furthermore, additional data from a control city where no similar electronic reminders were inserted into the EHR system would have improved interpretation of the observed outcomes. We are not able to describe the process by which electronic reminders produced the effect they did on PCPs’ recording of diagnosis since there was no possibility of carrying out an additional questionnaire survey.

Finally, we have no data concerning individual physicians and their behavior. Therefore, we cannot draw conclusions about whether there were physicians who did not respond to this intervention. For the same reason, we cannot exclude the possibility that there may have been physicians who regularly recorded inappropriate diagnoses despite the electronic reminders. At this point, it must also be emphasized that despite the increase in recorded diagnoses with electronic reminders, categorizing patients by means of diagnoses per se does not automatically lead to “better treatment” of these patients.^30^ All the observed diagnoses are not necessarily recorded,^31^ and all the recorded diagnoses are not necessarily adequate with respect to the patient’s medical condition, as has been suggested to be the case in about 15% of the PCP-consultations.^32^ Thus, while enhancing the quality of treatment from the health care system’s point of view, the present activity does not necessarily improve the quality of care experienced by the patients.^1^

## Conclusion

Electronic reminders enhance the extent of recorded diagnosis data in office-hours primary care practices in the PCHCs. When applied for the present purpose, electronic reminders may also influence the relative proportions of various recorded diagnoses. They were found to be effective in enhancing the recording rate of diagnoses related to chronic diseases and symptomatic diagnoses. Electronic reminders may be useful primers in primary health care when attempting to change the behavior of PCPs.
